# Identification of *WUSCHEL-related homeobox* gene and truncated small peptides in transformation efficiency improvement in *Eucalyptus*

**DOI:** 10.1186/s12870-023-04617-w

**Published:** 2023-11-30

**Authors:** Zhuo-Ao Zhang, Mei-Ying Liu, Shu-Ning Ren, Xiao Liu, Yue-Hao Gao, Chen-Yu Zhu, Hao-Qiang Niu, Bo-Wen Chen, Chao Liu, Weilun Yin, Hou-Ling Wang, Xinli Xia

**Affiliations:** 1 State Key Laboratory of Tree Genetics and Breeding, National Engineering Research Center of Tree Breeding and Ecological Restoration, College of Biological Sciences and Technology, Beijing Forestry University, Beijing, 100083 China; 2 Guangxi Key Laboratory of Superior Timber Trees Resource Cultivation, Guangxi Forestry Research Institute, 23 Yongwu Road, Nanning, Guangxi 530002 China

**Keywords:** *WUSCHEL-related homeobox* (*WOX*), *Eucalyptus grandis*, Peptides, Transformation efficiency, *E. urophylla × E. grandis* DH32-29

## Abstract

**Background:**

The *WUSCHEL-related Homeobox* (*WOX*) genes, which encode plant-specific homeobox (HB) transcription factors, play crucial roles in regulating plant growth and development. However, the functions of *WOX* genes are little known in *Eucalyptus*, one of the fastest-growing tree resources with considerable widespread cultivation worldwide.

**Results:**

A total of nine *WOX* genes named *EgWOX1*-*EgWOX9* were retrieved and designated from *Eucalyptus grandis*. From the three divided clades marked as Modern/WUS, Intermediate and Ancient, the largest group Modern/WUS (6 EgWOXs) contains a specific domain with 8 amino acids: TLQLFPLR. The collinearity, cis-regulatory elements, protein-protein interaction network and gene expression analysis reveal that the WUS proteins in *E. grandis* involve in regulating meristems development and regeneration. Furthermore, by externally adding of truncated peptides isolated from WUS specific domain, the transformation efficiency in *E. urophylla* × *E. grandis* DH32-29 was significant enhanced. The transcriptomics data further reveals that the use of small peptides activates metabolism pathways such as starch and sucrose metabolism, phenylpropanoid biosynthesis and flavonoid biosynthesis.

**Conclusions:**

Peptides isolated from WUS protein can be utilized to enhance the transformation efficiency in *Eucalyptus*, thereby contributing to the high-efficiency breeding of *Eucalyptus*.

**Supplementary Information:**

The online version contains supplementary material available at 10.1186/s12870-023-04617-w.

## Background

Transcription factors are a group of protein molecules that bind specifically to gene regulatory regions to ensure that the target gene is expressed with a specific intensity at a specific time and space. They play a crucial role in regulating plant growth and development processes [1]. The *WUSCHEL-related homeobox* (*WOX*) is a family of plant-specific transcription factors. It is characterized by the presence of a short stretch of which containing 60–66 conserved amino acid residues that fold into a DNA-binding domain called the homeodomain, which is encoded by the HB DNA sequence [2, 3]. The homeodomain binds to DNA through a helix-turn-helix (HTH) structure, which is characterized by two α-helices [3]. The *WOX* family is distinguished by its phylogenetically related homologous structures [4]. Based on evolutionary relationships, WOX members can be divided into three clades: the Modern/WUS clade, the Intermediate clades, and the Ancient clades [5]. The *WUS* gene is the earliest gene identified in the *WOX* gene family [6]. In lower plants such as green algae, vascular-free moss, and *Physcomitrella* patens, only one of the three clades of *WOXs* exists, namely the Ancient clade [7, 8]. The Intermediate clade emerged in the ferns and is related to the vascular plants, whereas *WOXs* in higher plants, such as seed-bearing gymnosperms and angiosperms, are present in all three clades [7, 8]. Nevertheless, it is believed that all three evolutionary branches are equally ancient, depending only on their function and selective adaptation [8]. Currently, a number of monocotyledonous and dicotyledonous plants have been successively identified as containing transcription factors with WUSCHEL-related homeobox domains. The *WOX* gene family was first discovered in *Arabidopsis thaliana* [9]. In *Arabidopsis*, the *WOX* gene family is consists of 15 members: *AtWUS* and *AtWOX1-7* belong to the WUS/Modern clade, *AtWOX8*, *9*, *11*, and *12* are classified into the Intermediate clade, and *AtWOX10*, *13*, and *14* are grouped into the Ancient clade.

Recent studies have shown that the WOX genes play crucial roles in important developmental processes, such as stem cell maintenance, embryonic patterning, and organ development in plants [6, 10–12]. Moreover, they also regulate the growth of shoot and root tip meristematic tissues and the balance of stem cells [4, 13, 14]. It was found that the expression of *WUS* in the ovule, shoot apical meristem, and anther makes a significant contribution to promoting central homogeneity in indeterminate buds and determinate floral meristems, as well as ovule development [4, 15–18]. The Modern/WUS genes appear to function in a similar manner that is related to stem cells [19–23]. To illustrate, *CLV3* and *WOX4* are expressed in the shoot apical meristem (SAM), while *WOX5* is expressed in the root quiescent center (QC) cells, which are surrounded by the stem cells. All of these genes play crucial roles in maintaining stem cells in *Arabidopsis* [19–22]. Furthermore, the intermediate and ancient clade genes primarily function in differentiation and development. For example, *WOX8* is expressed in the fertilized egg to ensure the proper development of the embryo [11]. *WOX9* is involved in maintaining normal cell proliferation and preventing premature differentiation in the SAM [24]. *WOX13* shows strong expression in mature flowers and young siliques, and it plays a role in fruit development [25]. *WOX14* is mainly expressed in vascular tissue and is involved in preventing premature differentiation of root and floral organs [26, 27].

*Eucalyptus* is one of the widely distributed fast-growing trees with significant economic value, characterized by rapid vegetative growth for biological biomass [28]. *Eucalyptus* is one of the most important sources of carbon sinks, bioenergy, and raw wood materials [29, 30]. The *Eucalyptus* species, which belong to the *Myrtaceae* family of angiosperms, are cultivated in tropical and subtropical areas around the world, and are the main source of woody biomass on earth [31]. Due to its rapid growth rate, adaptability to various ecological conditions, and high-quality wood fiber, *Eucalyptus* is the primary hardwood for pulp and wood production [32]. The *Eucalyptus* species also contribute to energy production and the recovery of pulping chemicals in paper mills through combustion, due to their high calorific value [33]. In addition, certain secondary metabolites of *Eucalyptus*, such as essential oils, can also serve as natural insecticides, holding great significance for human daily life [31].

While the key structure and developmental functions of the *WOX* gene family have been extensively studied in model plants such as *Arabidopsis*, there is a lack of research on woody plants, with very limited studies available. Based on the functions of WOX protein in various species, we hypothesized that WOX protein may have the potential to enhance transformation efficiency in woody plants. Based on the functions of the WOX protein in multiple species, we hypothesized that the WOX protein may have the potential to promote transformation efficiency. In our study, we identified nine *Eucalyptus WOX* genes (*EgWOXs*) and analyzed their phylogeny, gene structure, physicochemical properties of the protein, cis-acting elements of promoter regions, expression profile in different plant tissues, and Protein–Protein Interaction (PPI) network. We also investigated the impact of truncated WOX peptides, identified from conserved motifs of WOX proteins, on the transformation efficiency of the *Eucalyptus urophylla* × *E. grandis* DH32-29 genotype. The results of this study have laid the foundation for researching the WUS peptide signaling pathway and enhancing the efficiency of *Eucalyptus* gene transformation.

## Results

### Identification, characteristics, phylogenetic, structure and protein conserved domain analyses of *WOX* genes in ***E. grandis***

Nine candidate *WOX* genes were identified in *E. grandis*. According to the position on the chromosome, gene names are arranged in ascending order. The length of the *EgWOX* gene ranged from 865 bp (*EgWOX7*) to 3,641 bp (*EgWOX8*) (Supplementary Table 1). The physicochemical properties of the*WOX* genes are presented in Supplementary Table 1. The protein length encoded by *EgWOXs* ranges from 181 amino acids (EgWOX7) to 368 aa (EgWOX2). The relative molecular weights of EgWOXs ranged from 20.429 kDa (EgWOX7) to 41.056 kDa (EgWOX2), and the protein isoelectric point varied between 6.64 (EgWOX1) and 10.01 (EgWOX9). Most proteins had isoelectric points higher than seven. Subcellular localization analysis revealed that all WOX proteins were localized in the nucleus (Supplementary Table 1).

**Fig. 1:**
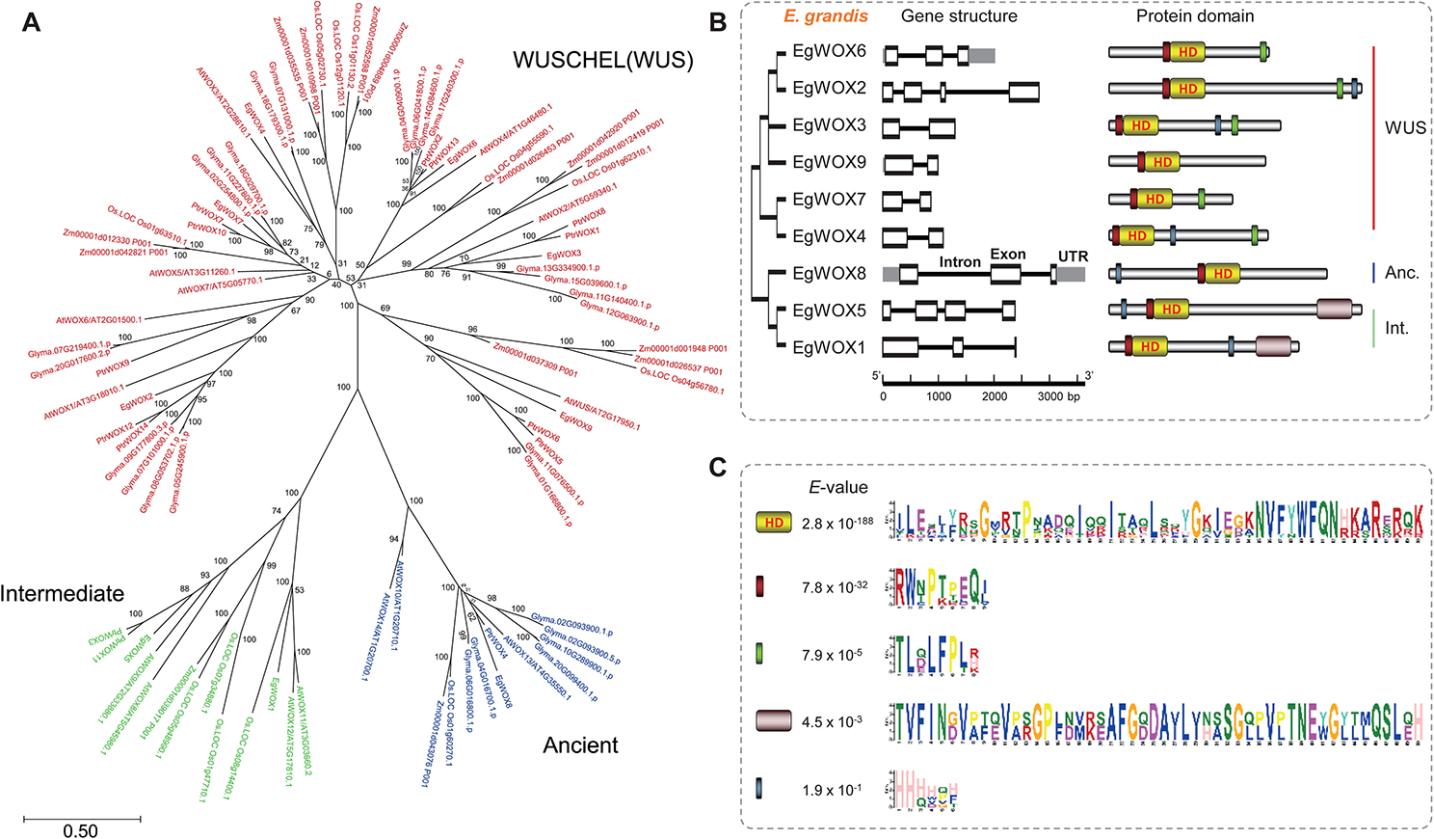
Identification of *WUSCHEL-related homeobox* genes in *E. grandis*. (**A**) Phylogenetic tree analysis of WOX proteins from *P. trichocarpa*, *E. grandis*, *A. thaliana*, *G. max*, *Z. mays*, and *O. sativa*. (**B**) Depiction of gene structure and protein domains of each WOX in *Eucalyptus*. The yellow rectangles represent the homeodomain (HD), and green rectangles represent the WUSCHEL (WUS)-box motif. (**C**) Sequence logos of the protein domains presented in *E. grandis*

A comprehensive unrooted phylogenetic tree was constructed to reveal the evolutionary and developmental relationships of the *WOX* family genes among *Populus trichocarpa*, *E. grandis*, *A. thaliana*, *Glycine max*, *Zea mays*, and *Oryza sativa*. As demonstrated in Fig. 1, the classification information of the *AtWOX* gene family shows that the *WOX* gene family can be primarily divided into three clades: WUSCHEL, Intermediate, and Ancient. The *WOXs* were unevenly distributed among the groups. The Modern/WUSHEL group contains 65 *WOX* genes, including six *EgWOX* genes (*EgWOXs 2, 3, 4, 6, 7*, and *9*), making it the largest group among *WOX* genes. The Intermediate group included 13 *WOX* genes, two of which were *EgWOX* genes (*EgWOX1* and *EgWOX5*). The Ancient group consisted of 13 *WOX* genes, including *EgWOX8*, making it the smallest group of *WOX* genes in *E. grandis* (Fig. 1A).

The gene structure of the *WOX* gene family was found to be relatively conserved within the same clade and diverse across different clades. By analyzing the structure of *WOX* genes, we found that all *EgWOX* genes consist of 2–4 exons. Most *EgWOX* genes contain two exons. All genes with only one intron belong to the modern/WUS clade. Only *EgWOX6* (modern/WUS clade) and *EgWOX8* (Ancient clade) contain untranslated regions (UTRs) (Fig. 1B).

To delve deeper into gene evolution, we conducted an analysis of the conserved regions of the protein. Our findings revealed that all WOX proteins contained motif 1, which encodes the Homebox Domain (HD), while all WUS branches included a protein domain characterized by TLQLFPL. All Intermediate clades also contain unique conserved regions (Fig. 1C). The variation in motifs among WOX proteins may play a significant role in their functional divergence [34–36]. Moreover, Pfam tool was also applied for domain screening and the results displayed in Supplementary Table 2 showed that conserved homeodomains were identified in all WOX proteins.

### Chromosome and synteny analysis

**Fig. 2:**
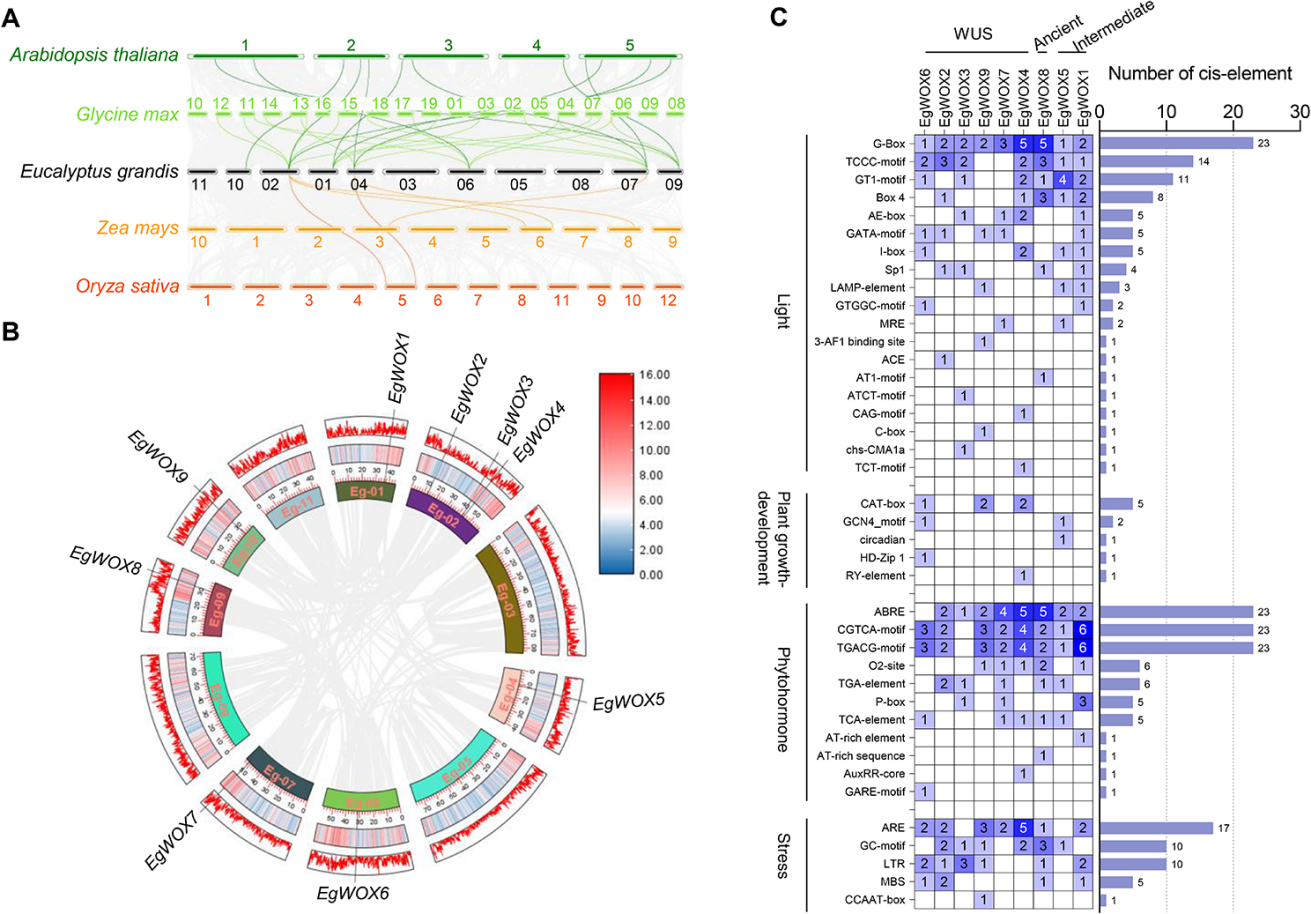
Synteny analysis of *WOX* genes in *E. grandis* and other four plant species. (**A**) The synteny analysis of *E. grandis* with *A. thaliana*, *G. max*, *Z. mays*, and *O. sativa*. The synteny blocks are shown in grey lines while the collinearity of *WOX* gene pairs are marked in color lines from four species. (**B**) Synteny analysis of *WOX* genes in *E. grandis*. Circle represents the chromosomes with different labels. The gene density of the chromosome was displayed in the outermost layer of the circle. (**C**) The gradient colors and histogram show the number of *cis*-elements in the promoter of *WOX* genes in *E. grandis*. The functional notes were enriched into four categories: light, plant growth development, phytohormone and stress

The analysis of the *WOX* genes in the genome revealed their uneven distribution across the chromosomes. The *EgWOXs* were distributed on seven chromosomes (Eg-01, 02, 04, 06, 07, 09, 10) (Fig. 2A and B). The uneven distribution of *WOX* genes among chromosomes indicates differences in chromosome size and structure within the genome.

To explore the evolutionary relationship of the *WOX* gene family, we conducted synteny analysis with four other species (*A. thaliana, G. max, O. sativa, and Z. mays*) and collinearity analysis within *E. grandis*. Gene replication events, including tandem and segmented replication, can effectively increase the abundance of gene families. Chromosome regions containing less than 200 kb of two or more genes were considered to be tandem duplication events [29]. For interspecific collinearity analysis, the chromosomal location analysis revealed differences in the distribution of *WOX* genes between two monocots and two dicots. Furthermore, each chromosome possessed a different number of *WOXs*. The collinear diagram illustrates that the *EgWOXs* had 12 pairs of isogenous genes in *Arabidopsis*, 18 pairs in soybean, 4 pairs in maize, and 2 pairs in rice (Fig. 2A). Since *Arabidopsis*, soybean, and *Eucalyptus* are dicotyledonous plants, the number of homologous *WOX* genes is much greater than that in monocotyledonous plants such as maize and rice.

To further investigate the natural evolutionary events of the *WOX* gene family, intraspecific collinearity analysis revealed no segmental duplicate gene pairs was found in *E. grandis* (Fig. 2B). This indicates that both segmental and tandem duplication events contribute to increasing genetic diversity during the evolutionary process of *EgWOXs*, which are relatively conservative.

### ***Cis*-acting element analysis of* EgWOX* promoter sequences**

To further investigate the potential regulatory mechanisms of the *WOX* gene family in light response, plant growth and development, phytohormones, and stress response, we identified and analyzed promoter *cis*-elements (Fig. 2C). We identified 89 *cis*-acting elements linked to light response in all candidate *EgWOX* genes, indicating a potential association between *WOX* gene expression patterns and the levels of *WOX* genes with light signals. In the analysis of plant growth and development, a CAT-box element related to the regulation of meristem expression was found in *E. grandis*. Moreover, the RY-element involved in seed-specific regulation, the GCN4-motif involved in endosperm, and the CAT-box involved in circadian control were also identified in *E. grandis*. Four common elements (TGA-element, O_2_-site, P-box, and GARE-motif) have also been found in relation to plant hormone regulation. These elements are involved in auxin response, zein metabolism, and gibberellin. In addition, elements associated with other phytohormones have also been found in *E. grandis*, namely TGACA-motif and CGTCA-motif (which are associated with MeJA-responsiveness), and ABRE (which is related to the responsiveness of abscisic acid). The results above suggest that the *EgWOXs* likely have various crucial physiological functions.

### WOX-related protein-protein interaction network

**Fig. 3:**
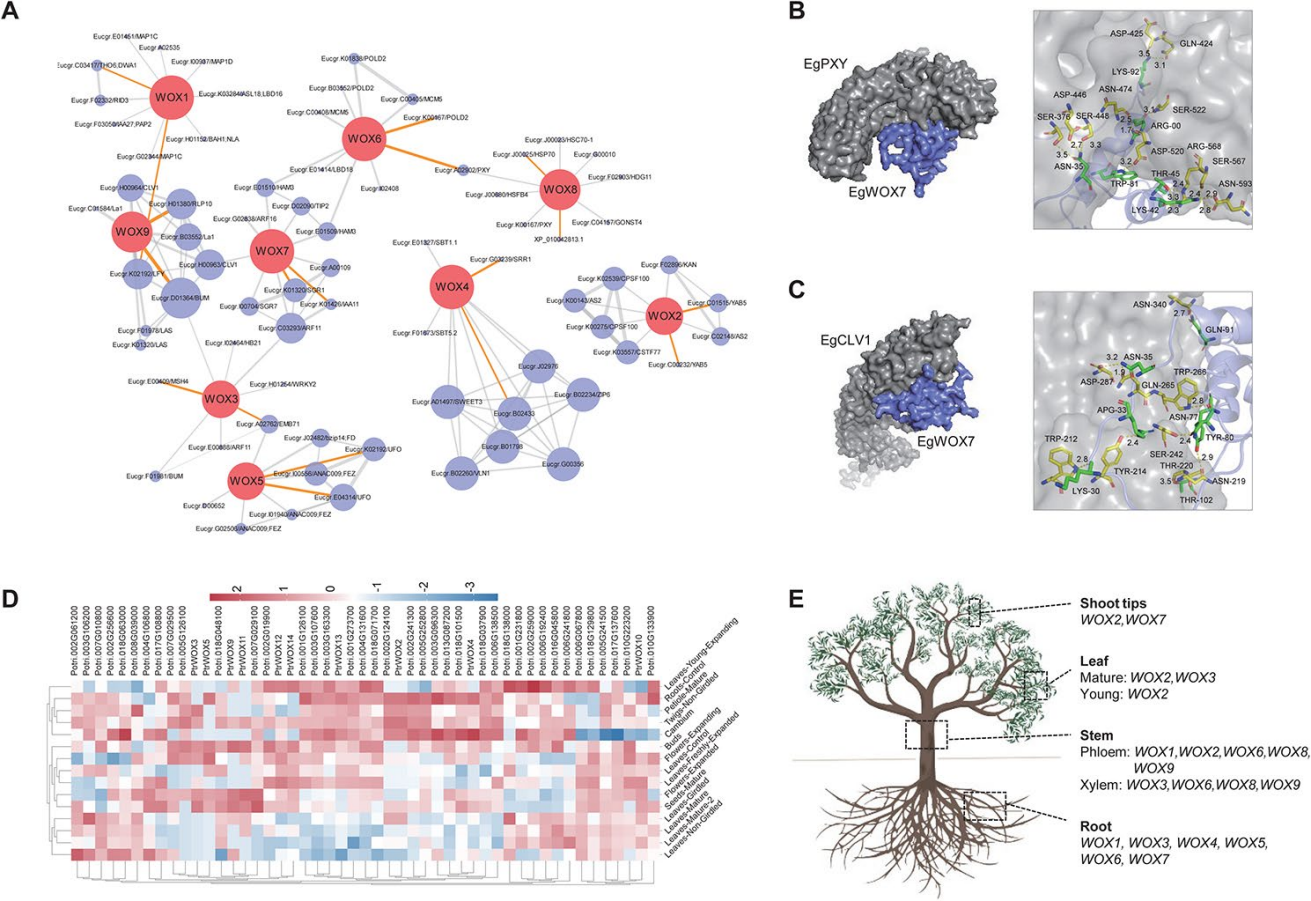
Protein-protein interaction networks and the expression pattern of *WOX* genes in *E. grandis*. (**A**) The PPI networks of WOX with other proteins in *E. grandis*. The correlations were downloaded from STRING database, and the network was constructed using Cytoscape v3.7.1 software. (**B**, **C**) The visualization of EgWOX7 interacts with EgPXY (**B**) or EgCLV1 (**C**) in *E. grandis*. The WOX protein was displayed in blue and the EgPXY or EgCLV1 proteins were indicated in grey. The ASN-452 means the 452th asparagine, and the number 3.5 means 3.5 angstrom between ASN-452 and LYS-92. (**D**) The expression features of *WOX* genes and their interaction genes in different tissues of *E. grandis*. The color bars indicate the log-transformed expression value. (**E**) The visualization expression pattern of *WOX* genes in tree model of *E. grandis*

In *E. grandis*, the EgWOX2 and EgWOX4 proteins were relatively independent branches. In general, proteins involved in similar signal transduction or metabolic pathways interact with each other. Therefore, interaction networks can effectively and intuitively predict gene patterns and multiple functions. The interaction network diagram of WOX proteins in *E. grandis* revealed that numerous proteins containing CLAVATA3/EMBRYO-SURROUNDING REGION (CLE), CLAVATA (CLV), POLD, PHLOEM INTERCALATED WITH XYLEM (PXY), and other proteins were predicted to interact with WOXs (Fig. 3A, C). The detailed interacting sites between EgWOX7 with EgPXY or EgCLV1 were visualized using the molecular docking tool (Fig. 3B, C). It was reported that CLE signals intersect with WUSCHEL-RELATED HOMEOBOX (WOX) functions and play a role in stem cell maintenance together [37–39]. CLV is closely related to the formation of the shoot apical meristem (SAM), and a molecular network called the CLAVATA (CLV)-WUSCHEL (WUS) pathway transmits signals that are critical for shoot and floral meristem development between cells [19]. POLD is associated with DNA replication and cell division in stem cells [40]. A study showed that dynamic gene regulation involves a feed-forward loop consisting of auxin, transcription factors (WUS), and PXY [41, 42]. PXY defines a procambium active region to maintain the vascular arrangement of the stem and regulate xylem cell development in the inflorescence stem [27, 41]. The analysis of the protein interaction network map predicted that the WOX protein is closely related to cell division, meristem formation, and differentiation in plants.

### **Expression of* EgWOX* genes in different tissues**

To investigate the role of *WOX* genes in organ development, gene expression data for various tissues of *E. grandis* were accessible for download from the website (https://plantgenie.org/) and visualized using TBtools v1.128 [19]. The expression data of *WOX* genes in the root, tip, leaf, xylem, phloem, flower, bud, and seed provide the basis for further study on the temporal and spatial expression. The results showed that all the downloaded *WOX* genes were constitutively expressed in a spatiotemporal-specific manner (Fig. 3D and E), revealing that *WOX* genes have various functions in different tissues.

**Fig. 4:**
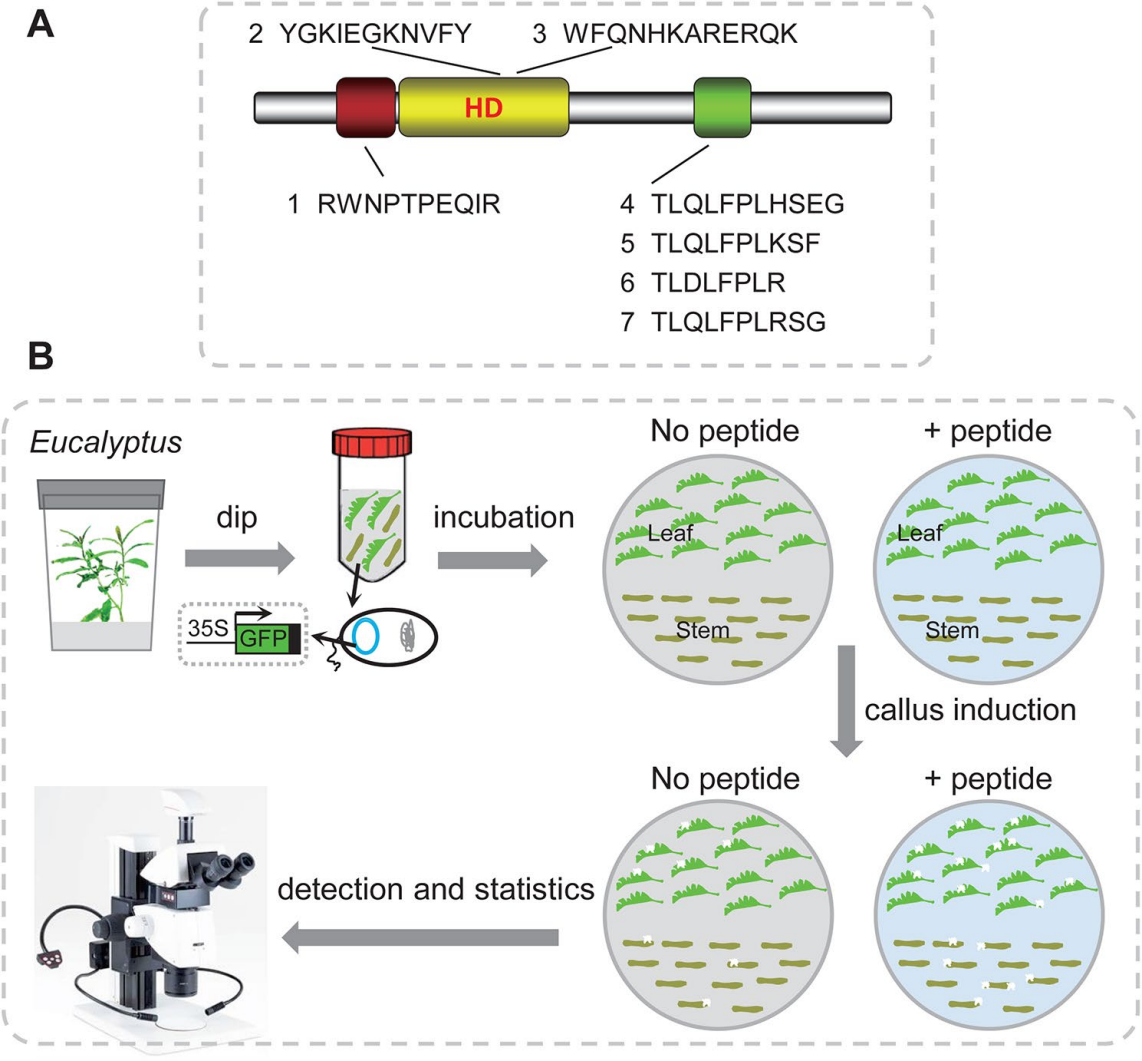
Overview of the transformation-efficiency-enhance based on peptide treatment from WOX protein. (**A**) Seven kinds of peptide were displayed from the WOX protein. Peptide 4, 5, 6, 7 were designed from the WUSCHEL (WUS)-box specific motif, and peptide 2, 3 were designed from the homeodomain. (**B**) Overview of the peptide treatment and transformation efficiency detection procedure. The *Agrobacterium* harboring the MAS::eGFP plasmid were used for genetic transformation. Seven kinds of peptide were added separately into the co-incubation and callus-induction media

### Exogenous adding of WOX peptide improves the transformation efficiency in *E. urophylla × E. grandis* DH32-29

**Fig. 5:**
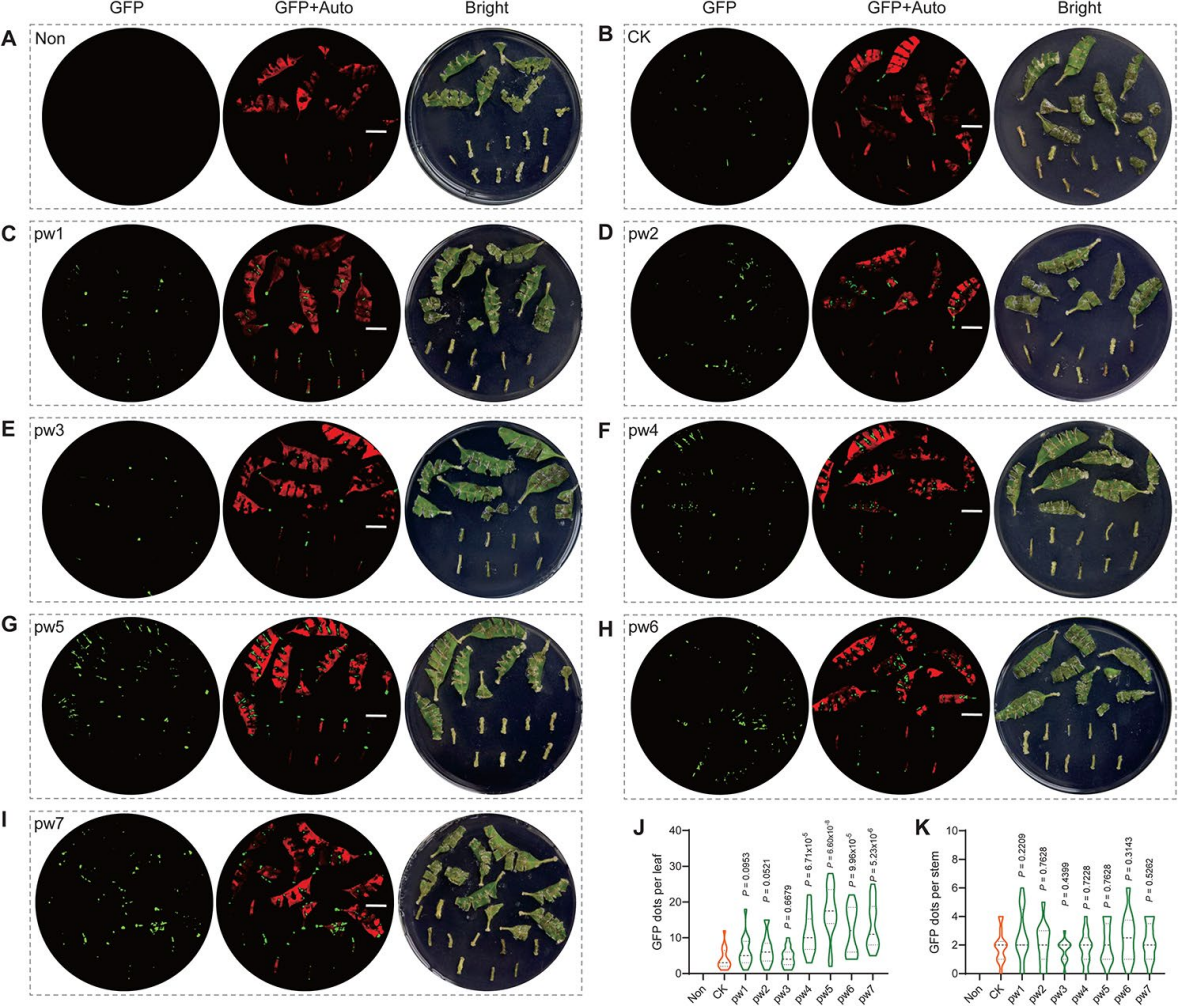
Effects of exogenous WOX peptide treatment on genetic transformation efficiency in *E. urophylla* × *E. grandis* DH32-29 genotype. (**A**-**H**) Extra addition of different peptide in the media. ‘Non’ means leaves and stems without *A. tumefaciens* infection, and ‘CK’ means infected with *A. tumefaciens* but without adding peptide. (**J**-**K**) Statistics of GFP dots per tissue in leaf (**J**) and stem (**K**)

*Agrobacterium*-mediated transformation (AMT) is the most commonly used biotechnology for modern plant genome engineering. However, achieving efficient AMT in the economically valuable species *Eucalyptus* remains a challenge, despite the continuous efforts of many research groups. We aimed to investigate whether the efficiency of AMT in *Eucalyptus* could be enhanced through the co-treatment of small peptides truncated from WOX protein domains. We designed seven peptides extracted from the sequence alignment of WOX proteins in *Eucalyptus*. Peptides 4, 5, 6, and 7 are derived from the WUSCHEL (WUS)-box motif, while peptides 2 and 3 are designed based on the homeodomain, and peptide 1 is derived from a conserved domain of WOX (Fig. 1C, and Fig. 4A). The schematic diagram of the entire assay was also displayed (see Fig. 4B). One-month-old leaves and stems cut from *E. urophylla* × *E. grandis* DH32-29 were infected with *Agrobacterium tumefaciens* strain EHA105 harboring pSUPER1300 plasmid, and co-incubated with or without various WOX peptides. The number of transgenic calli produced with GFP signal, which can be detected with a fluorescence microscope, was counted (Fig. 5). The leaves and stems without *A. tumefaciens* infection were used as negative control which marked as ‘Non’, while the tissues infected with *A. tumefaciens* but without adding peptide were marked as ‘CK’. The results show that the addition of pw1, pw2, or pw3 has no significant effect on the transformation efficiency, while the exogenous addition of pw4, pw5, pw6, and pw7 promotes the transformation efficiency. The average number of GFP dots per leaf in the control group (CK) is 4.1. In the experimental groups, the statistics data for pw4, pw5, pw6, and pw7 were 10.9, 17.3, 11.9, and 13.4, respectively (Fig. 5J). However, there are no significant differences in stems when exogenously adding WOX peptide.

### Transcriptome analysis and KEGG pathway enrichment analysis after small peptide treatment

**Fig. 6:**
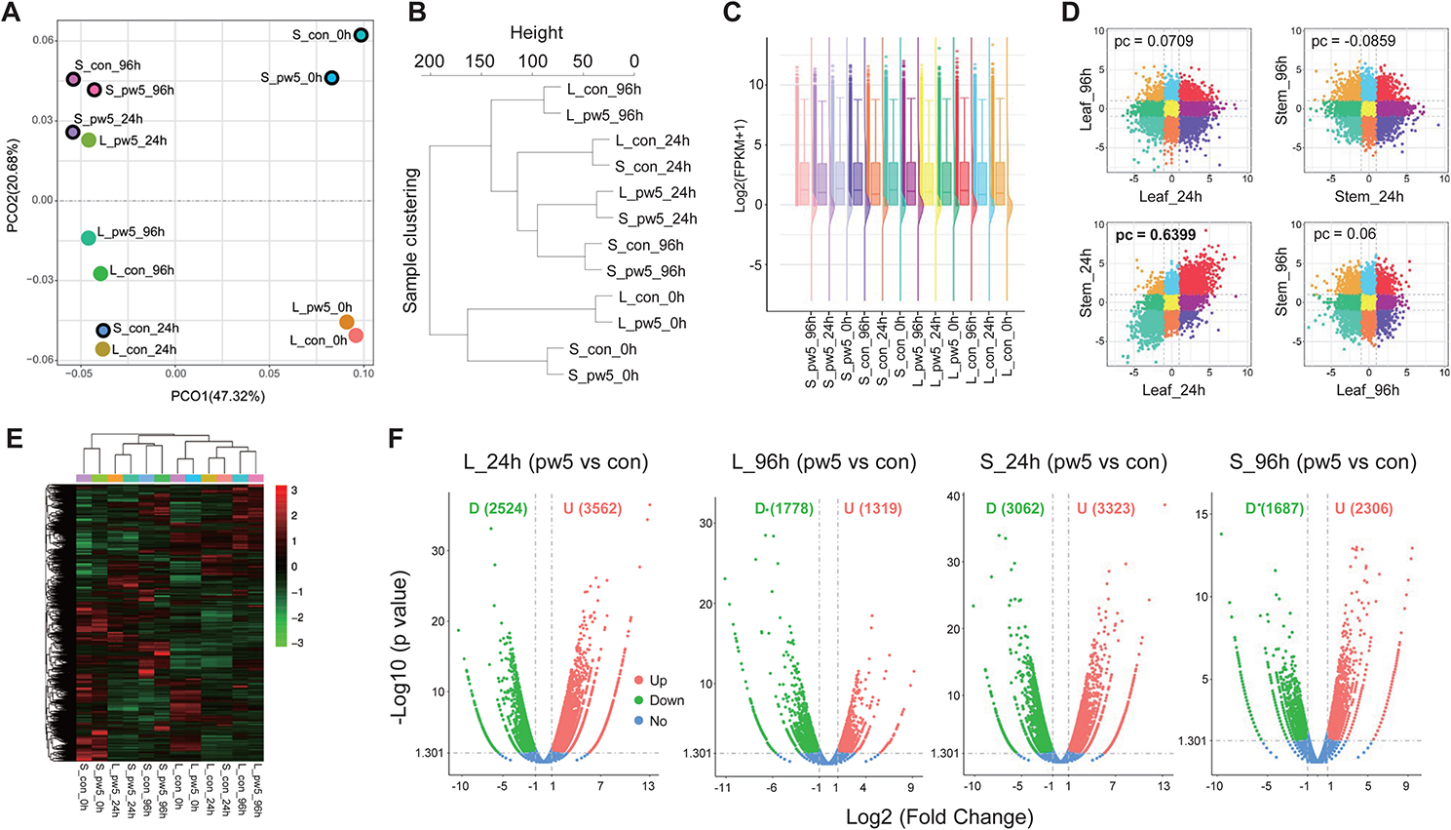
Transcriptomic analysis of the exogenous WOX peptide treatment on leaves and stems in *E. urophylla* × *E. grandis* DH32-29 genotype. (**A**) Principal coordinate analysis (PCoA) of the transcriptome analysis of leaf and stem after WOX peptide treatment. Each data point refers to one time point of sampling from 5 individual plants. S, stem; L, leaf; con, control/CK; pw5, WOX peptide 5. (**B**) Sample clustering analysis. (**C**) Cloud-rain diagram shows discreteness of the FPKM values. The expression values were log_2_ (FPKM + 1) transformed. (**D**) Nine-quadrant diagram shows the correlation between different samples. pc, Pearson Correlation. (**E**) Cluster analysis of DEGs in all samples. (**F**) DEGs in different combination. D, down-regulated; U, up-regulated

To investigate the mechanism by why exogenously added peptides promote transformation efficiency, we conducted a transcriptome analysis following treatment with peptide pw5. The principal coordinate analysis (PCoA) (Fig. 6A) and hierarchical clustering (Fig. 6B) were used to arrange samples based on their overall similarity or dissimilarity. The results showed that the pattern of the transcriptomes varied across samples, explaining 47.32% of the variation along the first principal component axis (Fig. 6A). The hierarchical clustering results were consistent with the PCoA results and confirmed that the samples clustered based on the treatment time (Fig. 6B). The discreteness of the FPKM values was illustrated by a cloud-rain diagram (Fig. 6C). The similarity in expression between different samples after peptide treatments was further analyzed using the Pearson Correlation (PC) method. The results indicated that after 24 h of pw5 treatment, the leaves and stems exhibited highly similar expression patterns, with a PC value of 0.6399 (Fig. 6D). Moreover, the differentially expressed genes (DEGs) between the samples were identified (Fig. 6E, F). After 96 h of treatment with pw5, the number of DEGs in the leaf was fewer than that of the 24-hour treatment, with 1,319 DEGs up-regulated and 1,778 DEGs down-regulated. The similar pattern was observed in the stems, and the results indicate that the exogenous addition of pw5 induces endogenous gene expression at the early stage.

**Fig. 7:**
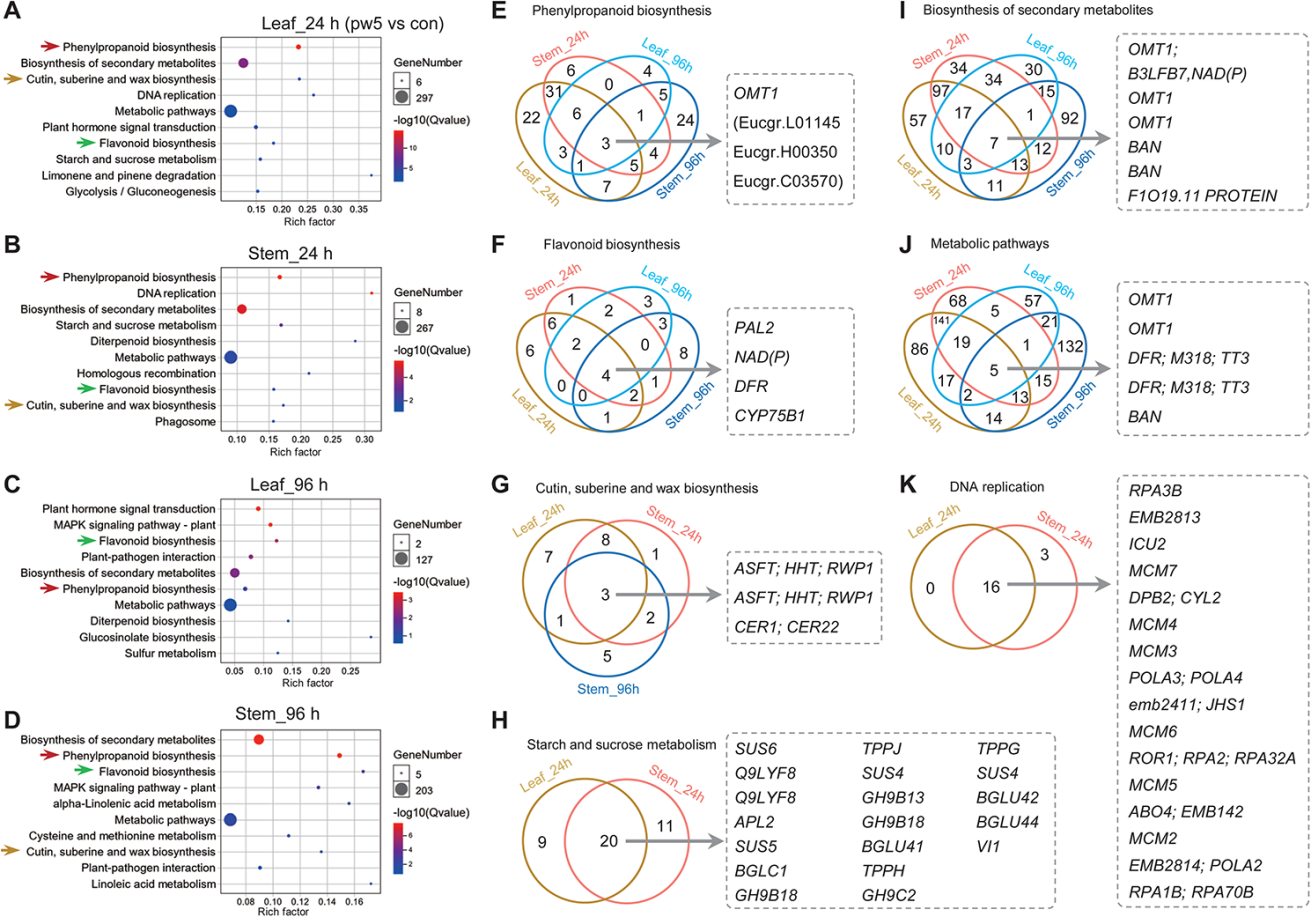
KEGG enrichment analysis of DEGs. (**A**-**D**) KEGG enrichment analysis of DEGs in leaf (**A**, **B**) and stems (**C**, **D**) after peptide treatment. Arrows show the same enriched pathways. (**E**-**H**) Venn diagram analysis of DEGs in different KEGG pathways of phenylpropanoid biosynthesis (**E**), flavonoid biosynthesis (**F**), cutin, suberine and wax biosynthesis (**G**), starch and sucrose metabolism (**H**), biosynthesis of secondary metabolites (**I**), metabolic pathways (**J**), and DNA replication (**K**). The name of the genes were displayed on the right of the venn diagram

To investigate the signaling pathways following pw5 treatment, we conducted KEGG pathway enrichment analysis using the DEGs (Fig. 7). In the leaves after 24 h of treatment with pw5, the up-regulated DEGs were mainly enriched in phenylpropanoid biosynthesis, biosynthesis of secondary metabolites, and cutin, suberin, and wax biosynthesis pathways (Fig. 7A). The result shows similarity with that in the stems after 24 h of treatment of pw5 (Fig. 7B). Interestingly, in both leaves and stems after 96 h of pw5 treatment, the DEGs were primarily enriched in phenylpropanoid biosynthesis and flavonoid biosynthesis (Fig. 7C, D). In general, treatment of plants with pw5 solution in leaves and stems induces the expression of O-methyltransferase 1 (OMT1), which is involved in regulating phenylpropanoid biosynthesis, the biosynthesis of secondary metabolites, and metabolic pathways (Fig. 7E, I, J). Moreover, phenylalanine lyase 2 (*PAL2*) and aliphatic suberin feruloyl-transferase (*ASFT*) are the key pw5-induced genes involved in regulating flavonoid biosynthesis and cutin, suberin, and wax biosynthesis, respectively (Fig. 7F, G). The minichromosome maintenance complex gene (*MCH*), which regulates DNA replication, and SUCROSE SYNTHASE 6 (*SUS6*), which regulates starch and sucrose metabolism, are also key genes induced by pw5.

### WOX peptide-induced genes are closely associated with metabolism

**Fig. 8:**
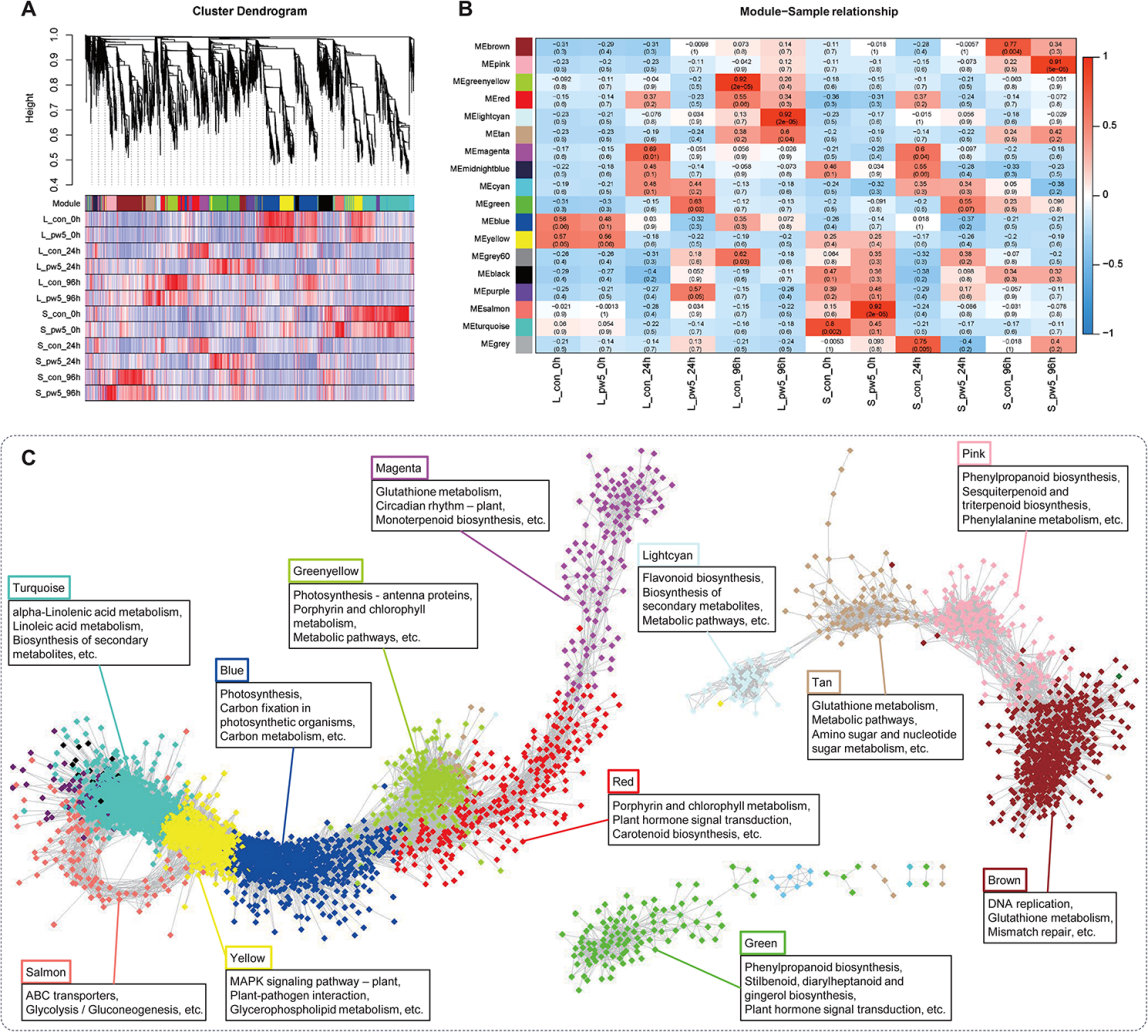
Transcriptomic correlation analysis of pw5 treatment across different time points. (**A**) Dendrogram showing co-expression modules (clusters) identified by WGCNA across different time points after pw5 treatment. Modules labeled with different colors were displayed in tree branches. (**B**) Module-sample correlations. Heatmap showing the correlation between identified modules and different leaf and stem samples. (**C**) Correlations network between 12 modules and KEGG enrichment of different modules

To screen and identify the hub genes involved in promoting transformation efficiency after pw5 treatment, we conducted weighted gene co-expression network analysis (WGCNA) using all differentially expressed genes (DEGs). The number of genes in each module ranged from 9 to 1,381. In leaf samples, the WGCNA analysis revealed four modules that were positively correlated with the pw5 treatment, including “greenyellow” and “lightcyan”, both with a correlation of 0.92 (p = 2.0 × 10^− 5^) (Fig. 8A, B). The “greenyellow” module is positively associated with the metabolism of photosynthesis antenna proteins, porphyrin, and chlorophyll (Fig. 8C). The “lightcyan” module is positively correlated with flavonoid biosynthesis, the biosynthesis of secondary metabolites, and metabolic pathways (Fig. 8C). The “green” module (correlation = 0.63, p = 0.03) was highly enriched in phenylpropanoid biosynthesis (Fig. 8C), which was consistent with the KEGG enrichment analysis of DEGs after 24 h of treatment with pw5 (Fig. 7A). Moreover, the “magenta” module (correlation = 0.69, p = 0.01), which displayed down-regulated genes after 24 h of pw5 treatment, was strongly associated with glutathione metabolism, circadian rhythm-plant, and monoterpenoid biosynthesis. In summary, these results suggest core physiological processes in the leaves and stems after pw5 treatment, indicating a positive correlation between gene expression and metabolic pathways.

**Fig. 9:**
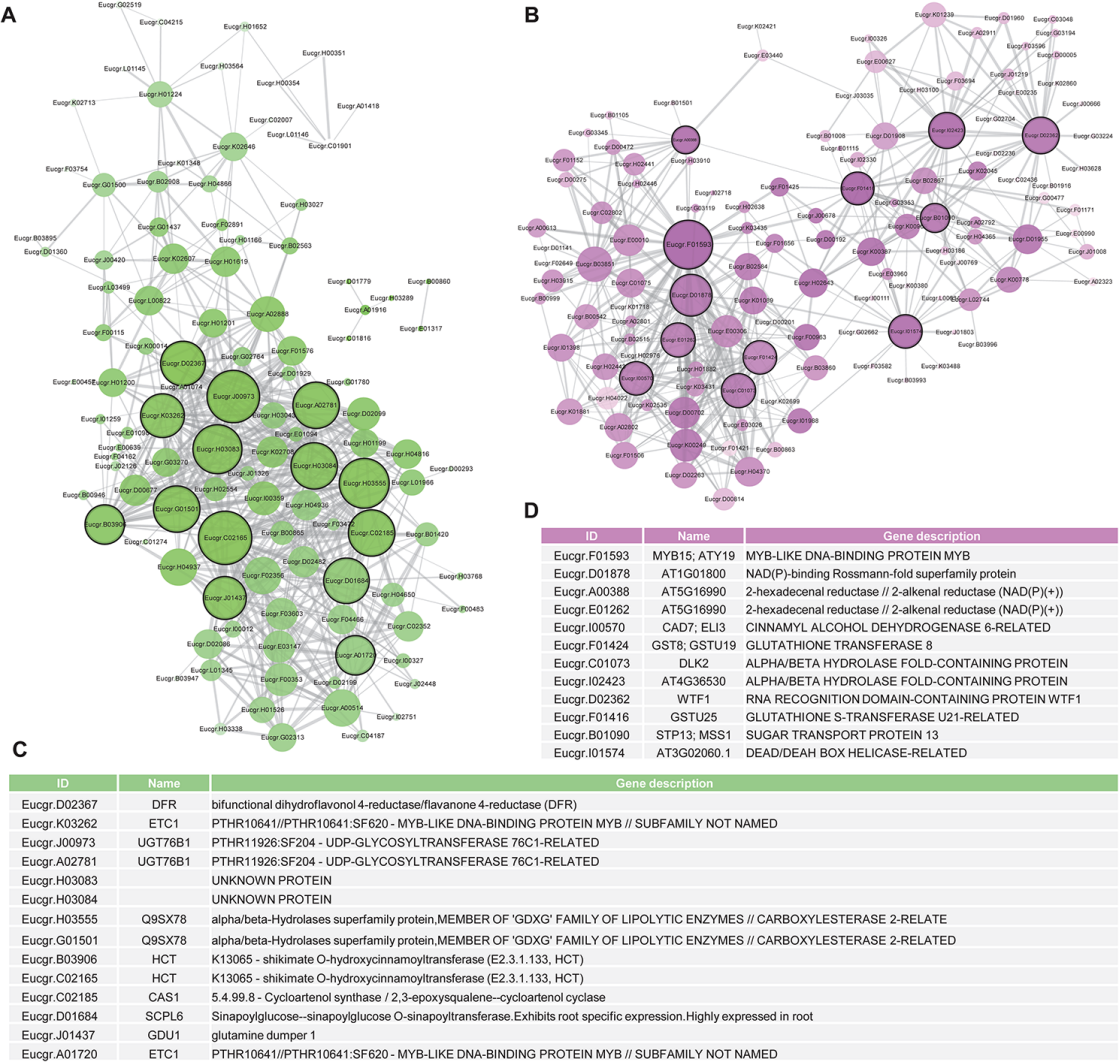
The correlation network of “green” and “magenta” module. (**A**, **B**) Correlation network showing the “green” (**A**) and “magenta” (**B**) module from Fig. 8. Gene pairs with the edge weight higher than 0.30 are kept and visualized. The thickness of the line indicates correlation value between two genes, and larger circles marked with black border mean hub genes. (**C**, **D**) The annotation of hub genes in “green” (**A**) and “magenta” (**B**) module

Furthermore, to identify the hub genes induced by pw5 in regulating transformation efficiency, we chose the two representative “green” and “magenta” modules to construct correlation networks. The network was analyzed using Cytoscape v3.7.1, and gene pairs with an edge weight higher than 0.30 were retained and visualized (Fig. 9). In the “green” module, 14 genes with larger circles, such as *HCT* (Eucgr.B03906), *DFR* (Eucgr.D02367), *ECT1* (Eucgr.K03262), and *UGT76B1* (Eucgr.J00973), were identified as hub genes (Fig. 9A, C). In the “magenta” module, a MYB-LIKE DNA-BINDING PROTEIN, *MYB15* (Eucgr.F01593), was identified as one of the hub genes

## Discussion

Although significant progress has been made in genetic transformation technology in many plant species over the past decade [43], *Eucalyptus*, which are currently recognized as the most important forestry plantation trees, exhibit very low transformation efficiency with high genotype dependence [44]. The situation limits the potential for improving *Eucalyptus* through transgenic and genome-editing integration approaches. Regeneration-related genes such as *WUS* and *BBM* have been shown to improve plant transformation efficiency in several species. However, manipulation of these factors often results in growth defects or teratogenesis due to the persistent expression of these developmental genes.

The *WOX* gene family is highly conserved and crucial in plants [45]. As concluded, the role of the *WOX* gene in cell division and differentiation in plant growth and development has been widely reported. Recently, genome-wide studies on the *WOX* gene family have been conducted and reported in various plants, including oil palm, banana, and apple [15, 46, 47]. The *WOX* gene family contains three clusters based on the motifs: WUSCHEL (WUS), Intermediate, and Ancient. This classification is consistent with that of other species, such as *Arabidopsis*, woodland strawberry, soybean, and wheat [45, 48, 49]. The application of WOX peptide primarily depends on the WUS-related protein domain.

The *WUS* genes with the (WUS)-box motif are widely studied and have been proven to be important regulators of somatic embryogenesis. In this study, we identified 9 *EgWOX* genes, including 6 *WUS* members in *E. grandis* (Fig. 1B). They were dispersed on different chromosomes, consistent with previous studies [34, 50, 51]. The number of introns is similar in both the Ancient and Intermediate clades of *WOX* family genes, as previously reported in banana and *C. sinensis* plants [47]. Analysis of both gene structure and conserved protein domains indicated that *WOX* genes within the same branch tended to exhibit similar structures and patterns across multiple species, demonstrating a close evolutionary relationship among *WOX* genes within each subfamily [52].

The number of WOX homologous genes in *E. grandis* is higher in dicotyledonous plants than in monocotyledonous plants, due to *Eucalyptus* being classified as a dicotyledonous plant. In addition, the number of collinearity events with soybean was significantly higher than that with *Arabidopsis*. This indicates a strong relationship between *A. thaliana*, soybean, *Populus*, and *Eucalyptus*. Furthermore, neither type of *WOX* gene replication event occurred in the *Eucalyptus* genome, and the occurrence of this phenomenon is worth exploring. By analyzing *cis*-elements of *WOX* genes, it was found that *E. grandis* is responsive to light (G-BOX, BOX4), plant growth and development (CAT-box, GCN4-motif), plant hormones (O_2_-site, ABRE), and stress (ARE) [53]. Similar findings were also observed in apple, tea plant, and *C. sativus* [15, 54, 55]. Most of the responsive elements are light-responsive, suggesting that optical signals likely play a role in regulating *WOX* gene expression.

A previous attempt to express a type III secretion system (T3SS) from *Pseudomonas syringae*, and the effectors AvrPto, AvrPtoB, or HopAO1, individually delivered to suppress host defense responses, increased transformation efficiency. The increase in yield ranges from 250 to 400% in wheat, alfalfa, and switchgrass [56]. Moreover, overexpression of the maize *BBM* and *WUS2* genes leads to higher transformation frequencies. Transgenic calli were recovered from over 40% of the starting explants, demonstrating the great potential of the *WUS* gene in increasing plant transformation efficiency [57]. However, in both methods, engineering *A. tumefaciens* or transformation vectors is necessary. In our study, we used a simple, convenient, and easy method to increase transformation efficiency in *E. urophylla × E. grandis DH32-29*. With our strategy of adding small peptides of truncated WUS proteins (pw5), we achieved up to 4.3 times more transformed calli compared to plants that did not receive the peptide treatment (Fig. 5). The limitation of this strategy is similar to the *A. tumefaciens* engineering method in which certain plant species or variety lines are resistant to regeneration, causing the method to lose efficacy. Nevertheless, it makes sense to investigate whether small peptides can promote regeneration, as WUS is essential for the de novo establishment of the shoot stem cell niche [58]. The high transformation efficiency is crucial for genome editing technology in plants, particularly in important agroforestry species such as *Eucalyptus*. To determine whether the enhanced transformation efficiency observed in our study is a result of altered signaling pathways, we examined the expression pattern following WUS peptide treatment. In the present study, after 24 h of treatment with pw5, the DEGs are highly enriched in starch and sucrose metabolism, as well as plant hormone transduction, which is consistent with previous reports in wheat [59]. The WUS clade genes have been shown to be involved in regulating plant regeneration [58, 60–62]. The application of regeneration-related genes could potentially enhance plant transformation efficiency during the in vitro culture media process [57, 63–69]. Carbohydrate metabolism is essential for plant callus induction and shoot regeneration [70]. Cambial cell division activity can be regulated by *WUSCHEL-RELATED HOMEOBOX 4* (*WOX4*)-like genes [71]. In our study, the application of small peptides isolated from the WUS protein activates metabolic pathways such as starch and sucrose, leading to enhanced regeneration in the callus of *E. urophylla* × *E. grandis* DH32-29, and contributes to an enhancement in transformation efficiency. In general, our method avoids the drawbacks of overexpressing developmental factor genes that promote transformation.

## Conclusion

Nine *EgWOX* genes have been identified and categorized into *WUS*, Intermediate, and Ancient clades. All EgWOX proteins were typical of a homeodomain. Promoter analysis indicates that WOXs are involved in regulating light, plant growth and development, phytohormones, and stress response. Most WOX proteins form a tightly interconnected network of interactions. The gene expression profiles in different tissues showed that WOX expression in *E. grandis* was spatially and temporally specific. The transformation experiments involving truncated peptides in *E. grandis × E. urophylla* DH32-29 indicated that WOX peptides could serve as signal peptides to facilitate plant transformation.

## Methods

### Plant materials and growth conditions

*E. urophylla* × *E. grandis* DH32-29 plants were cultivated on half-strength Murashige & Skoog (MS) medium containing 1% sucrose and 0.6% agar with 2.0 mg/L of 3-Indolebutyric acid (IBA) and 100 mg/L of L-Ascorbic Acid Sodium Salt, in the growth chambers (24 °C; 16 h light/8 hours dark).

### Identification of *WUSCHEL-related homeobox (WOX)* genes in *E. grandis*

The genome sequences and annotation files of *E. grandis* v2.0 were downloaded from the Phytozome 13.0 database (https://phytozome-next.jgi.doe.gov/). We identified all members of the *WOX* family by retrieving the Hidden Markov model of the *WOX* structural domain (PF00046) from the Pfam database (http://pfam.xfam.org/) and searching the genome of *E. grandis* using the Blast Compare Two Seqs program in TBtools v1.128 software [72, 73]. Gene identification (IDs) of the predicted *EgWOX* gene family were further confirmed using the Protein BLAST function on the National Center for Biotechnology Information (NCBI, https://www.ncbi.nlm.nih.gov/) database. Accordingly, all the selected *WOX* genes were searched in the Phytozome 13.0 database [74], and relative information, such as genome, CDS, transcripts, polypeptides, and promoter sequence was extracted.

### Phylogenetic analysis of *WOX* family genes

The WOX genes from four other species (*A. thaliana, G. max, Z. mays, and O. sativa*) were identified from PlantTFDB (http://planttfdb.gao-lab.org/) [75]. The *WOX* gene sequences were aligned using the ClustalX tool [76], and visualized with MEGA11 software (https://www.megasoftware.net/) [77]. Subsequently, the intraspecific phylogenetic trees of *E.grandis*, and the interspecific phylogenetic tree of six species were established using MEGA11 via the Neighbor-Joining (NJ) method with bootstrap analysis and the Jones-Taylor-Thornton (JTT) model based on 1000 replicates [77].

### Analysis of gene structures and conserved domains

The gene structures were determined using genomic and CDS sequence, and then illustrated using the online software Gene Structure Display Server (http://gsds.cbi.pku.edu.cn/) [78]. To identify conserved motifs, the amino acid sequences of *EgWOXs* were analyzed using the online MEME server (http://meme-suite.org/) [79]. The top five motifs with the lowest *E* values were displayed.

### Analysis of cis-regulatory elements in promoter of *EgWOX* genes

To identify *WOX* gene promoters, the 2000 bp upstream of the translation start sites were analyzed using the PlantCARE database (http://bioinformatics.psb.ugent.be/webtools/plantcare/html/) [80]. The data was visualized using TBtools v1.128 software [72].

### Collinearity selection pressure calculation analysis of *WOX* genes

The interspecies and intraspecies syntenic analyses were conducted using the protein sequences and the One Step MCScanX program in TBtools v1.128, with an E-value threshold of 10^− 10^ for BlastP [72]. The collinearity analysis results were visualized using the Dual Synteny Plotter and Advanced Circos program in TBtools v1.128 software.

### Construction of protein–protein Interaction Network

The online STRING tool (https://cn.string-db.org/) was used to analyze protein–protein interactions (PPI) [81], and the interaction information was then visualized in Cytoscape v3.7.1 (https://apps.cytoscape.org/) [82]. The amino acid sequences of each WOX protein in *E. grandis* were uploaded to the STRING website. The default settings were applied, and the TSV file displaying only one-way edges (A-B) was downloaded. The node1 and node2 were used as ‘node’ while the ‘combined score’ was used as ‘edge’ for image visualization in Cytoscape software, and the thickness of the line indicates the edge value between two proteins, thicker line means higher correlation value and and larger circles means more correlated nodes. The subcellular localization of the WOX family genes was determined using the Plant-mPLoc server (http://www.csbio.sjtu.edu.cn/bioinf/plant-multi/) [83–85].

### Molecular docking for protein interaction visualization

The protein structures of EgWOX7 and its interacting proteins EgPXY and EgCLV1 were generated using the I-TASSER server (https://zhanggroup.org/) for homologous template modeling, which automatically identifies template proteins [86]. After obtaining the protein structure, we used the HDOCK Server (http://hdock.phys.hust.edu.cn/) for protein docking. The strongest weakly polar interaction force points were modified and marked as stick structures, and the hydrogen bonds were indicated by a yellow dashed line. Only the hydrogen bonds with a length of 4.0 or less were displayed. The receptor protein (EgPXY or EgCLV1) was transformed into a surface form, while the ligand protein (EgWOX7) was transformed into a cartoon form [87].

### **Genetic transformation with exogenous WOX peptide treatment in* E. urophylla × E. grandis* DH32-29**

The truncated proteins were synthesized at Sangon Biotech (Shanghai). For the transformation of *Eucalyptus* with WOX peptide, the cut stem segments and young leaves of *E. urophylla* × *E. grandis* DH32-29 were pre-cultured on a 0.6% agar medium containing Murashige & Skoog (MS), 30 g/L sucrose, 0.5 mg/L 6-Furfurylaminopurine (Kinetin, KT), 1.0 mg/L 2,4-Dichlorophenoxyacetic acid (2,4-D), and 100 mg/L L-ascorbic acid sodium salt (VC Na) for 2 days. Afterwards, the leaves and stem were dipped into a suspension of *A. tumefaciens* bacteria (EHA105) carrying the pSUPER1300 vector for 15 min (lightly shaking the suspension every 2–3 min), and cultured in the co-incubation medium (MS media with 30 g/L sucrose, 0.5 mg/L KT, 1.0 mg/L 2,4-D, 100 mg/L VC Na) with or without 0.5 µM peptide for 4 days. The leaves and stems were then transferred to selection media containing 0.5 mg/L glyphosate ammonium (Basta) for 20 days to identify eGFP positive lines. The transformation results were confirmed using a fluorescence microscope and then statistically analyzed.

### RNA-Seq and transcriptomic data analysis

For each data point, leaf and stem samples were collected from 5 individual plants. RNA-Seq data were generated using an Illumina Novaseq6000 system at Novogene Ltd. (Beijing). The transcriptomic data analysis was conducted according to the methods described in the previous study [88]. Briefly, raw fastq format reads were trimmed and filtered using in-house Perl scripts. Subsequently, the clean reads were aligned to the *E. grandis* v2.0 genome using the Hisat2 algorithm [89]. To measure gene expression levels, the Fragments Per Kilobase of exon model per Million mapped fragments (FPKM) was applied, and then the differentially expressed genes (DEGs) were identified using the DESeq2 tool [90].

The heatmaps were generated using the pheatmap R package [91] and GraphPad Prism 9 software [92]. The Kyoto Encyclopedia of Genes and Genomes (KEGG) pathway enrichment analysis [54] was conducted using the method described previously [88]. The Venn diagram analysis was conducted using the online tool InteractiVenn (http://www.interactivenn.net/). The cloud-rain diagram was used to distribute FPKM values, the nine-quadrant diagram was used to calculate correlations between different samples, and the volcano diagram was used to showcase DEGs. These analyses were performed using the online data analysis and visualization tool, Omicshare (https://www.omicshare.com/).

The cluster dendrogram module, module-sample correlation, and gene co-expression analysis were conducted using the R package WGCNA [93] (https://horvath.genetics.ucla.edu/html/CoexpressionNetwork/Rpackages/WGCNA/). Hub genes in the selected modules were identified by calculating the expression similarity of gene pairs (edge weight) using the WGCNA method. Subsequently, network analysis and visualization were performed using Cytoscape v3.7.1 software (https://cytoscape.org/), and only edge weight values higher than 0.30 were retained and visualized.

### Electronic supplementary material

Below is the link to the electronic supplementary material.

**Supplementary Material 1**. WOX-related protein-protein interaction network information in *E. grandis*

**Supplementary Material 2**. **Supplementary Table 1**. Identification of WOX genes in *E. grandis*. **Supplementary Table 2**. The homeodomain of WOX proteins identified using Pfam tool in *E. grandis*

## Data Availability

The datasets used and/or analysed during the current study are available from the corresponding author on reasonable request. The raw RNA-Seq data have been uploaded and deposited in the Sequence Read Archive (SRA) database (https://www.ncbi.nlm.nih.gov/sra/) with the accession number PRJNA967503.
