# Supplementary material for: Identification of WUSCHEL-related homeobox gene and truncated small peptides in transformation efficiency improvement in Eucalyptus
Source: BMC Plant Biol. 2023 Nov 30;23:604. doi: 10.1186/s12870-023-04617-w (PMC10688041; doi:10.1186/s12870-023-04617-w)
Supplement: Supplementary file 2 — Supplementary Material 2. Supplementary Table 1. Identification of WOX genes in E. grandis. Supplementary Table 2. The homeodomain of WOX proteins identified using Pfam tool in E. grandis [file 12870_2023_4617_MOESM2_ESM.docx]

**Supplementary Table 1.** Identification of *WOX* genes in *E. grandis*.

**Supplementary Table 2.** The homeodomain of WOX proteins identified using Pfam tool in *E.grandis*.

| Gene name | Family | Description | Entry type | Clan | Envelope | | Alignment | | HMM | | HMM length | Bit score | E-value | Predicted active sites |
| --- | --- | --- | --- | --- | --- | --- | --- | --- | --- | --- | --- | --- | --- | --- |
|  |  |  |  |  | Start | End | Start | End | From | To |  |  |  |  |
| EgWOX1 | Homeodomain | Homeodomain | Domain | CL0123 | 24 | 85 | 25 | 85 | 2 | 57 | 57 | 47.5 | 1.30E-12 | n/a |
| EgWOX2 | Homeodomain | Homeodomain | Domain | CL0123 | 77 | 138 | 79 | 138 | 3 | 57 | 57 | 68.7 | 3.00E-19 | n/a |
| EgWOX3 | Homeodomain | Homeodomain | Domain | CL0123 | 10 | 71 | 12 | 71 | 3 | 57 | 57 | 65.2 | 3.80E-18 | n/a |
| EgWOX4 | Homeodomain | Homeodomain | Domain | CL0123 | 5 | 66 | 6 | 66 | 2 | 57 | 57 | 65 | 4.50E-18 | n/a |
| EgWOX5 | Homeodomain | Homeodomain | Domain | CL0123 | 55 | 116 | 56 | 116 | 2 | 57 | 57 | 54.8 | 6.70E-15 | n/a |
| EgWOX6 | Homeodomain | Homeodomain | Domain | CL0123 | 79 | 139 | 80 | 139 | 3 | 57 | 57 | 67.6 | 6.70E-19 | n/a |
| EgWOX7 | Homeodomain | Homeodomain | Domain | CL0123 | 30 | 91 | 31 | 91 | 2 | 57 | 57 | 65.3 | 3.60E-18 | n/a |
| EgWOX8 | Homeodomain | Homeodomain | Domain | CL0123 | 129 | 190 | 130 | 190 | 2 | 57 | 57 | 65.3 | 3.60E-18 | n/a |
| EgWOX9 | Homeodomain | Homeodomain | Domain | CL0123 | 41 | 103 | 42 | 103 | 2 | 57 | 57 | 62.5 | 2.70E-17 | n/a |
| EgWOX1 | GAGE | GAGE protein | Family | n/a | 76 | 134 | 79 | 121 | 5 | 47 | 107 | 10.9 | 5.90E-01 | n/a |
|  | Ribosomal_L32p | Ribosomal L32p protein family | Family | CL0167 | 77 | 114 | 77 | 101 | 4 | 27 | 56 | 10.1 | 7.90E-01 | n/a |
| EgWOX3 | DUF1523 | Protein of unknown function (DUF1523) | Family | n/a | 124 | 190 | 133 | 186 | 39 | 91 | 176 | 11.7 | 1.30E-01 | n/a |
| EgWOX4 | GTA_TIM | GTA TIM-barrel-like domain | Domain | n/a | 19 | 114 | 25 | 104 | 115 | 194 | 299 | 10.4 | 3.30E-01 | n/a |
| EgWOX5 | SUIM_assoc | Unstructured region C-term to UIM in Ataxin3 (shorten) | Disordered | n/a | 71 | 155 | 80 | 151 | 14 | 59 | 64 | 16.6 | 6.20E-03 | n/a |
|  | OmpH | Outer membrane protein (OmpH-like) | Domain | n/a | 78 | 160 | 82 | 155 | 39 | 103 | 140 | 13.1 | 9.30E-02 | n/a |
| EgWOX6 | RHD3_GTPase | Root hair defective 3 GTP-binding protein (RHD3) GTPase domain (shorten) | Domain | CL0023 | 119 | 199 | 130 | 198 | 20 | 83 | 245 | 11.3 | 1.80E-01 | n/a |
| EgWOX8 | Tra1_ring | Tra1 HEAT repeat ring region | Repeat | CL0020 | 1 | 245 | 2 | 167 | 1104 | 1243 | 1691 | 14.6 | 4.30E-03 | n/a |
|  | DUF2076 | Uncharacterized protein conserved in bac … | Family | n/a | 2 | 98 | 9 | 71 | 106 | 176 | 262 | 17 | 5.10E-03 | n/a |
|  | BPL_LplA_LipB | Biotin/lipoate A/B protein ligase family | Domain | CL0040 | 20 | 139 | 28 | 133 | 13 | 99 | 133 | 12.7 | 1.00E-01 | n/a |
